# Characterization of sulopenem antimicrobial activity using *in vitro* time-kill kinetics, synergy, post-antibiotic effect, and sub-inhibitory MIC effect methods against *Escherichia coli* and *Klebsiella pneumoniae* isolates

**DOI:** 10.1128/spectrum.01898-24

**Published:** 2025-02-05

**Authors:** Joshua M. Maher, Michael D. Huband, Jill M. Lindley, Paul R. Rhomberg, Steven I. Aronin, Sailaja Puttagunta, Mariana Castanheira

**Affiliations:** 1 Element Iowa City–JMI Laboratories, North Liberty, Iowa, USA; 2 Iterum Therapeutics, Old Saybrook, Connecticut, USA; Seton Hall University, South Orange, New Jersey, USA

**Keywords:** post-antibiotic effect, time-kill curves, synergism, susceptibility testing, urinary tract infection, antimicrobial agents

## Abstract

Sulopenem is an oral and intravenous penem antibiotic in clinical development for treatment of urinary tract and intra-abdominal infections caused by multidrug-resistant pathogens. This study evaluated *in vitro* antimicrobial activity of sulopenem by post-antibiotic effect (PAE), sub-inhibitory minimal inhibitory concentration PAE effect (PAE-SME), checkerboard testing, and time-kill testing. Testing sulopenem at 1×, 5×, or 10× the baseline MIC resulted in a PAE interval of 0.0–0.7 hours. When exposed to 0.5× the sulopenem MIC following 5× MIC, all isolate/agent combinations had PAE-SME values of >4.8 hours. Checkerboard testing revealed no instances of antagonism between sulopenem and comparator agents—indifference was observed in most sulopenem checkerboard combinations. Sulopenem demonstrated bactericidal activity (≥3 log_10_ [99.9%] reduction in viable organism counts) in all time-kill assays following 24 hours of incubation at 8× the baseline MIC (6/6), 5/6 displaying this activity within 8 hours. The present antimicrobial parameters seen at concentrations surrounding the MIC support optimization of sulopenem dosing and further development. The oral dosing regimen of sulopenem etzadroxil/probenecid 500 mg/500 mg administered every 12 hours was recently evaluated in two phase 3 clinical trials where sulopenem demonstrated efficacy against amoxicillin-clavulanate in uncomplicated urinary tract infection (uUTI) and against ciprofloxacin in fluoroquinolone-resistant uUTI.

**IMPORTANCE:**

Sulopenem is an oral and intravenous penem antibiotic in clinical development for treatment of urinary tract and intra-abdominal infections caused by multidrug-resistant pathogens. This study evaluated sulopenem via broth microdilution susceptibility testing, PAE, sub-inhibitory MIC PAE effect, checkerboard testing, and time-kill testing. The results of this study—interpreted along with recent pharmacodynamic *in vitro* one-compartment and hollow-fiber infection model work—provide insight into the *in vitro* activity of sulopenem.

## INTRODUCTION

Sulopenem, a novel penem antibiotic agent, offers a promising solution to the escalating threat of multidrug-resistant bacterial infections. This broad-spectrum agent distinctively possesses both oral and intravenous formulations, allowing for potential step-down antibiotic therapy (1). Sulopenem—approved by the US Food and Drug Administration for oral use on 24 October 20224—has recently completed several clinical trials focused on complicated (NCT03357614) and uncomplicated urinary tract infections (uUTIs) (NCT03354598 and NCT05584657), as well as intra-abdominal infections (NCT03358376). Importantly, the sulopenem spectrum of activity extends to include fluoroquinolone-resistant, extended-spectrum β-lactamase (ESBL)-producing and multidrug-resistant organism groups except for carbapenemase-producing isolates (2–5).

While the *in vitro* and pre-clinical efficacy of sulopenem have been documented (3, 5–8), a critical need to evaluate the antimicrobial attributes of this compound remains. This study utilized checkerboard, time-kill kinetics, post-antibiotic effect (PAE), and sub-inhibitory MIC effect (SME) methods to gain further insight into the *in vitro* activity of sulopenem. Ertapenem was included in time-kill, PAE, and PAE-SME testing to provide data on an additional penem agent alongside. Broth microdilution checkerboard testing assessed interactions between antibiotics, specifically, whether combination therapy yields indifferent, synergistic, or antagonistic interactions. Interactions are evaluated by calculating the fractional inhibitory concentrations (ΣFIC) based on the Loewe additivity zero-interaction theory (9, 10). The PAE and PAE-SME are additional parameters measured under *in vitro* conditions that can assist with decisions regarding dose and duration of antibiotic therapy. The PAE and PAE-SME provide insight into the suppression of bacterial growth following a finite period of exposure to the antibiotic agent (PAE) (11) or when re-exposed to the agent at sub-inhibitory concentrations (PAE-SME) (12). *In vitro* time-kill kinetic assays quantify the interaction between an antibiotic and a bacterial population, measuring the concentration-dependent bactericidal or bacteriostatic activity of the agent (13). These methods reveal distinct elements of sulopenem activity and provide relevant information for the continued development of this antibiotic agent.

## RESULTS

### Checkerboard testing

Indifference (ΣFIC >0.5 to ≤4) was observed with the majority of sulopenem checkerboard combinations (Tables 1 and 2); no instances of antagonism (ΣFIC >4) were observed. Synergy (ΣFIC ≤0.5) was observed with sulopenem in combination with trimethoprim-sulfamethoxazole for *Escherichia coli* ATCC 35218 (ΣFIC_min_ = 0.38) and *E. coli* 937054 (ΣFIC_min_ = 0.5) and with sulopenem in combination with gentamicin against *Klebsiella pneumoniae* 396798 (ΣFIC_min_ = 0.5; Tables 1 and 2). The ΣFIC and subsequent synergy/indifferent/antagonism categorizations could not be determined for 10 of the sulopenem-agent combinations tested against *E. coli* strains and for 8 of the sulopenem-agent combinations tested against *K. pneumoniae* strains, as the MIC value for the comparator agent was greater than the highest concentration tested (Tables 1 and 2). The highest ΣFIC_max_ value observed for any agent-organism combination was 2.5 (Table 1).

**TABLE 1:** Summary of the minimum and maximum fractional inhibitory concentration (ΣFIC) values for sulopenem and comparator agents when tested in checkerboard combinations against Enterobacterales isolates and strains^*a*^

Organism	Amoxicillin		Aztreonam		Ceftriaxone		Doxycycline		Gentamicin		Levofloxacin		Nitrofurantoin		Vancomycin		Trimethoprim-sulfamethoxazole	
	Min.	Max.	Min.	Max.	Min.	Max.	Min.	Max.	Min.	Max.	Min.	Max.	Min.	Max.	Min.	Max.	Min.	Max.
*E. coli* NCTC 13353	–^*b*^	–	–	–	–	–	1.02	1.50	0.63	1.25	0.75	1.25	1.03	1.50	–	–	–	–
*E. coli* ATCC 35218	–	–	0.63	1.25	0.56	0.75	0.75	1.25	0.52	1.01	0.56	1.25	0.56	1.25	0.75	≥1.25	**0.38**	0.56
*E. coli* ATCC 25922	0.63	1.25	0.75	1.25	0.75	1.25	1.13	1.50	0.51	0.75	0.75	1.25	0.56	1.25	–	–	0.75	1.25
*E. coli* 937054	–	–	–	–	–	–	0.56	0.75	0.52	1.06	0.504	1.25	0.52	1.25	0.52	≥1.13	**0.50**	1.13
*K. pneumoniae* ATCC 700603	–	–	–	–	0.52	1.13	1.004	2.50	1.06	2.13	1.06	1.50	0.504	1.25	–	–	0.63	1.25
*K. pneumoniae* 396798	–	–	–	–	–	–	0.51	0.75	**0.50**	0.63	0.502	1.25	–	–	–	–	0.51	1.13

^*a*^ Synergy values are listed in bold**.**

^*b*^ –, the ΣFIC was not determined.

**TABLE 2:** Categorical interpretations for sulopenem antimicrobial agent combinations when tested in checkerboard studies against Enterobacterales isolates and strains

Organism	Amoxicillin	Aztreonam	Ceftriaxone	Doxycycline	Gentamicin	Levofloxacin	Nitrofurantoin	Vancomycin	Trimethoprim-sulfamethoxazole
*E. coli* NCTC 13353	–^*a*^	–	–	Indifferent	Indifferent	Indifferent	Indifferent	–	–
*E. coli* ATCC 35218	–	Indifferent	Indifferent	Indifferent	Indifferent	Indifferent	Indifferent	Indifferent	Synergy
*E. coli* ATCC 25922	Indifferent	Indifferent	Indifferent	Indifferent	Indifferent	Indifferent	Indifferent	–	Indifferent
*E. coli* 937054	–	–	–	Indifferent	Indifferent	Indifferent	Indifferent	Indifferent	Synergy
*K. pneumoniae* ATCC 700603	–	–	Indifferent	Indifferent	Indifferent	Indifferent	Indifferent	–	Indifferent
*K. pneumoniae* 396798	–	–	–	Indifferent	Synergy	Indifferent	–	–	Indifferent

^*a*^ –, the ΣFIC was not determined.

### Post-antibiotic and sub-MIC effects

Sulopenem and ertapenem baseline broth microdilution MIC, PAE, and PAE-SME values for all six strains are presented in Tables 3 and 4. PAEs were observed when testing sulopenem at 5× or 10× MIC (0.2–0.7 hours) against ATCC 35218 and at 5× sulopenem MIC against the clinical isolate 396798 (PAE = 0.7 hours). For ertapenem, isolates had a PAE of 0.2–1.1 hours when testing at 1×, 5×, or 10× MIC. The longest PAE observed was 1.1 hours at the ertapenem 10× MIC condition against *K. pneumoniae* ATCC 700603. No PAE was observed for sulopenem against ATCC 25922, ATCC 700603, NCTC 13353, and the *E. coli* clinical isolate 937054. No PAE was observed for ertapenem against NCTC 13353. Generally, PAE-SMEs greater than 3.0 hours (>4.8 for ATCC 25922, 4.2 for ATCC 35218, 4.5 for NCTC 13353, 3.0 for 937054, 4.6 for ATCC 700603, and 3.7 for 396798) were observed when isolates were exposed to 5× the ertapenem MIC for 1 hour then subsequently challenged with sub-MIC concentrations (0.25×). Comparatively, lower PAE-SMEs were observed when isolates were exposed to 5× the sulopenem MIC for 1 hour then additionally challenged with sub-MIC (0.25×) concentrations; PAE-SME values were 0.9 for ATCC 25922, 1.7 for ATCC 35218, 1.0 for NCTC 13353, > 5.2 for 937054, 2.5 for ATCC 700603, and 4.3 for 396798. When exposed to 0.5× MIC following 5× MIC, all isolate/agent combinations had off-scale PAE-SME values >4.8 hours. These isolates failed to increase 1 log_10_ in CFU over the 7 hours of exposure to 0.5× MIC concentrations of ertapenem or sulopenem.

**TABLE 3:** Summary of the MIC values for sulopenem and comparator agents when tested via broth microdilution against Enterobacterales isolates and strains^*a*^

Antimicrobial agent	Modal MIC (mg/L)					
	*E. coli* NCTC 13353	*E. coli* ATCC 35218	*E. coli* ATCC 25922	*E. coli* 937054	*K. pneumoniae* ATCC 700603	*K. pneumoniae* 396798
Amoxicillin	>256	>256	4	>256	>256	>256
Aztreonam	**>64**	0.06	0.12	**>64**	**32**	**>64**
Ceftriaxone	**>64**	0.03	0.06	**>64**	**4**	**>64**
Doxycycline	4	1	0.5	1	**16**	8
Ertapenem	0.12	0.008	0.008	0.25	0.06	0.25
Gentamicin	**128**	0.5	0.5	0.5	4	**64**
Levofloxacin	**2**	0.03	0.016	**8**	0.5	**>32**
Nitrofurantoin	8	16	8	16	64	**>256**
Sulopenem	0.06	0.03	0.03	0.12	0.12	0.06
Trimethoprim-sulfamethoxazole	**>64**	0.25	0.06	0.06	1	1
Vancomycin	>256	>256	>256	>256	>256	>256

^*a*^ The modal MIC value was determined from triplicate MIC testing. Resistant MIC values per CLSI breakpoint criteria (M100, Ed34, 2024) are indicated in bold.

**TABLE 4:** Summary of sulopenem and ertapenem MIC, PAE, and PAE-SME results^*b*^

Organism	β-Lactamase content	Broth microdilution MIC^*a*^ (mg/L)	Broth macrodilution MIC^*a*^ (mg/L)	PAE (hours)			PAE-SME (hours)	
				1× MIC	5× MIC	10× MIC	5× to >0.25× MIC	5× to >0.5× MIC
*E. coli* ATCC 25922	β-Lactamase negative	SUL—0.03	SUL—0.03	0.0	0.0	0.0	0.9	>5.4
		ERT—0.008	ERT—0.015	0.0	0.5	0.0	>4.8	>4.8
*E. coli* ATCC 35218	TEM-1	SUL—0.03	SUL—0.03	0.0	0.2	0.7	1.7	>5.4
		ERT—0.008	ERT—0.015	0.0	1.0	1.0	4.2	>5.3
*E. coli* NCTC 13353	CTX-M-15 and OXA-1	SUL—0.06	SUL—0.06	0.0	0.0	0.0	1.0	>5.3
		ERT—0.12	ERT—0.25	0.0	0.0	0.0	4.5	>5.2
*E. coli* 937054	CTX-M-15	SUL—0.12	SUL—0.12	0.0	0.0	0.0	>5.2	>5.2
		ERT—0.25	ERT—0.25	0.4	0.4	0.8	3.0	>5.2
*K. pneumoniae* ATCC 700603	SHV-18 and OXA-2	SUL—0.12	SUL—0.25	0.0	0.0	0.0	2.5	>5.3
		ERT—0.06	ERT—0.12	0.9	0.9	1.1	4.6	>5.2
*K. pneumoniae* 396798	CTX-M-15, SHV-1, and OXA-1/30	SUL—0.06	SUL—0.12	0.0	0.7	0.0	4.3	>5.4
		ERT—0.25	ERT—0.5	0.9	0.2	0.2	3.7	>5.3

^*a*^ The modal MIC value was determined from triplicate MIC testing.

^*b*^ AE, post-antibiotic effect; ERT, ertapenem; MIC, minimum inhibitory concentration; PAE-SME, sub-inhibitory MIC effect; SUL, sulopenem.

### *In vitro* time-kill

**Fig 1:**
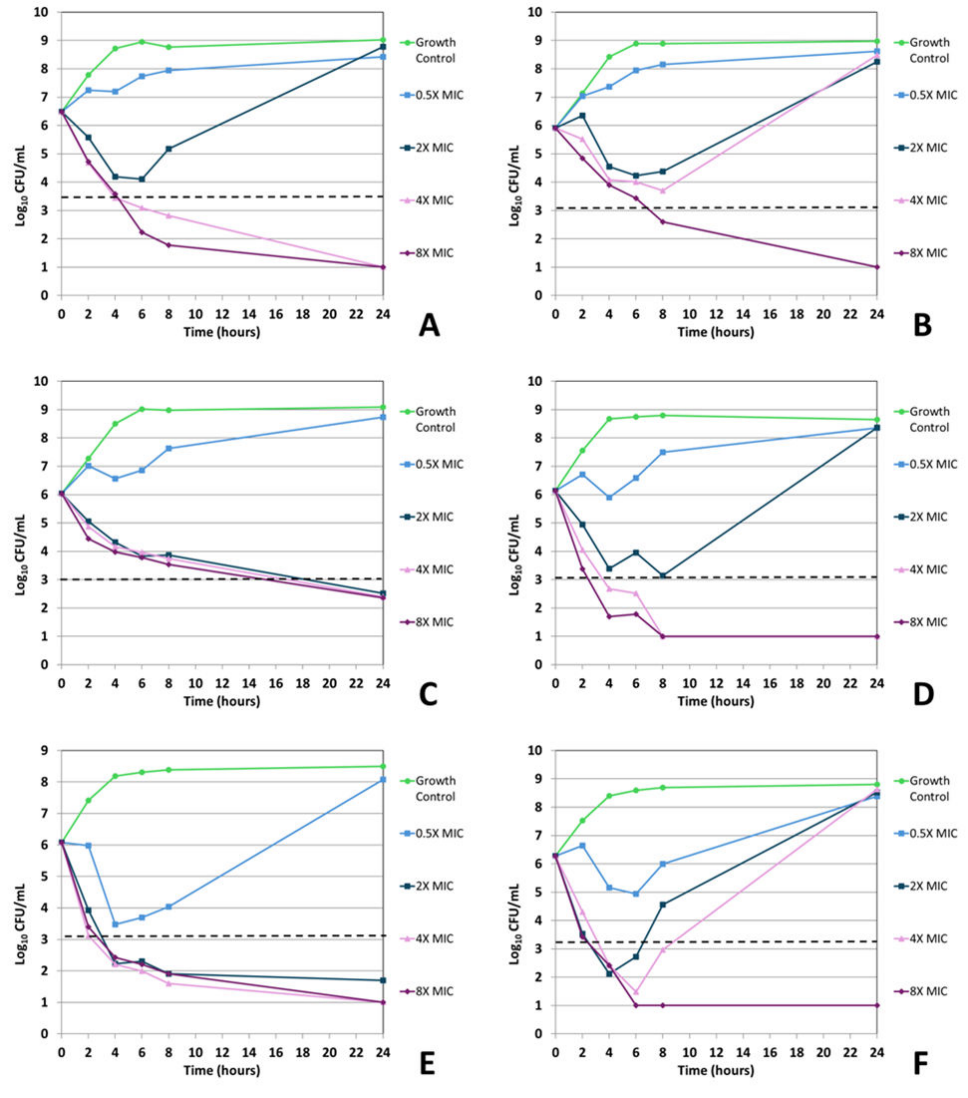
*In vitro* time-kill curve analysis of sulopenem against all isolates and strains. Time-kill curves for iteration 1 of sulopenem against *E. coli* NCTC 13353 (**A**), *E. coli* ATCC 25922 (**B**), *E. coli* ATCC 35218 (**C**), *E. coli* 937054 (**D**), *K. pneumoniae* 396798 (**E**), and *K. pneumoniae* ATCC 700603 (**F**). The dashed line indicates ≥3 log_10_ CFU/mL reduction in viable organism counts compared to the untreated growth control.

**Fig 2:**
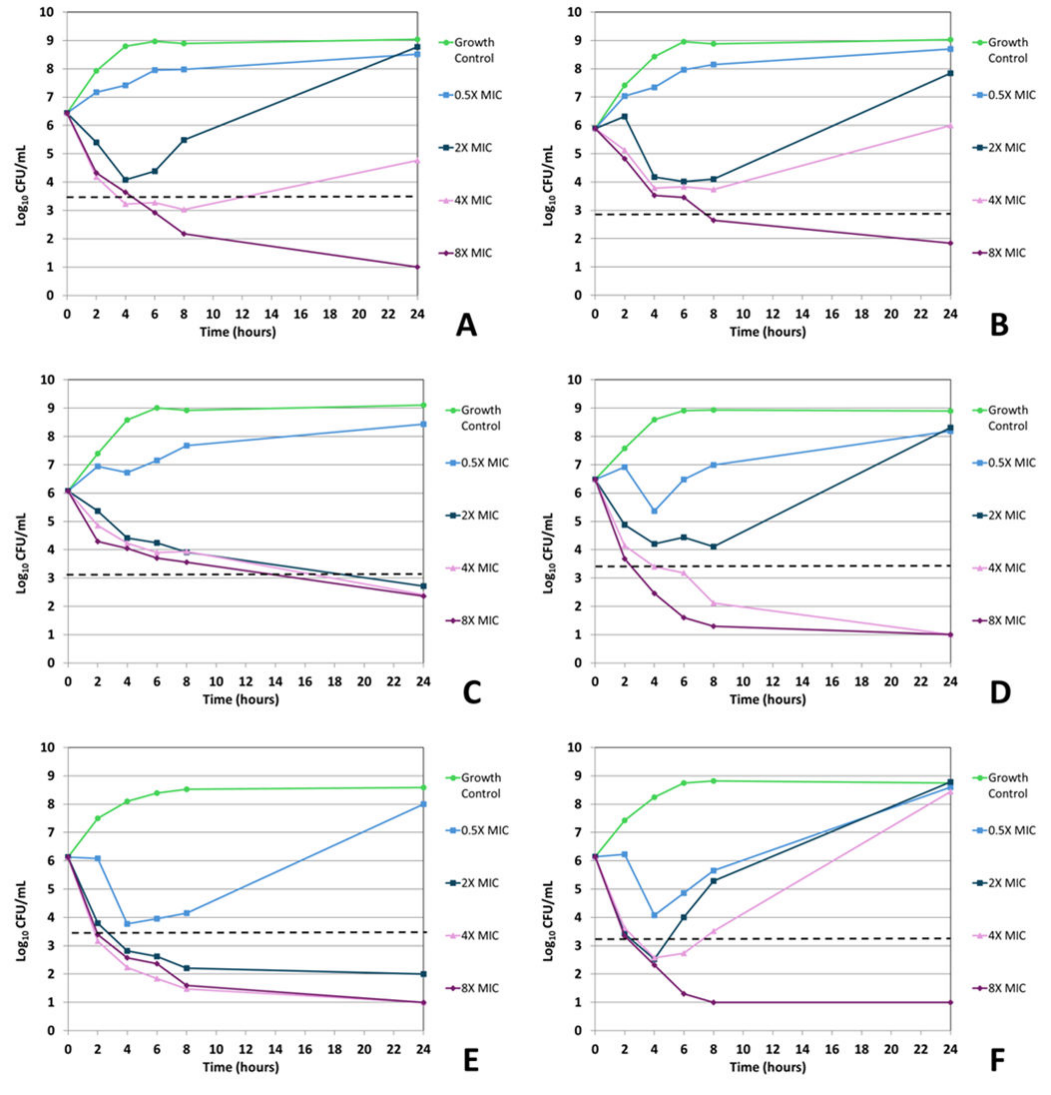
*In vitro* time-kill curve analysis of sulopenem against all isolates and strains. Time-kill curves for iteration 2 of sulopenem against *E. coli* NCTC 13353 (**A**), *E. coli* ATCC 25922 (**B**), *E. coli* ATCC 35218 (**C**), *E. coli* 937054 (**D**), *K. pneumoniae* 396798 (**E**), and *K. pneumoniae* ATCC 700603 (**F**). The dashed line indicates ≥3 log_10_ CFU/mL reduction in viable organism counts compared to the untreated growth control.

Sulopenem demonstrated bactericidal activity (≥3 log_10_ [99.9%] reduction in viable organism counts) in all time-kill assays after 24 hours of incubation at 8× the baseline MIC concentration (Fig. 1 and 2; Table 5). At 4× MIC, sulopenem displayed bactericidal activity against ATCC 35218, 937054, and in one repetition against NCTC 13353. At the 2× MIC concentration, two of the sulopenem-isolate time-kill assays demonstrated bactericidal activity after 24 hours (ATCC 35218 and 396798). All isolates grown at a sub-MIC sulopenem concentration (0.5× MIC) showed an increase in CFUs following 24 hour incubation (1.7–2.8 log_10_ increase; Table 5). Notably, of the 8× sulopenem time-kill conditions displaying a ≥3 log_10_ reduction in viable organism counts following 24 hours of incubation (6/6), 5/6 displayed this activity within 8 hours (Table 6).

**TABLE 5:** Summary of MIC and decrease in cell viability during the time-kill kinetics assay at the 24-hour time point for sulopenem and ertapenem against all isolates and strains^*b*^

Organism	Broth macrodilution MIC^*a*^ (mg/L)	Replication	Antimicrobial agent concentration (relative to MIC)			
			0.5×	2×	4×	8×
*E. coli* ATCC 25922	SUL—0.03	1	−2.7	−2.3	−2.6	**4.9**
		2	−2.8	−2.0	−0.1	**4.0**
	ERT—0.015	1	−2.4	**5.1**	**5.1**	**5.1**
		2	−2.0	**5.2**	**5.2**	**5.2**
*E. coli* ATCC 35218	SUL—0.03	1	−2.7	**3.5**	**3.6**	**3.7**
		2	−2.4	**3.4**	**3.7**	**3.7**
	ERT—0.015	1	**3.9**	**4.0**	**3.7**	**3.7**
		2	**3.9**	**3.8**	**3.5**	**3.7**
*E. coli* NCTC 13353	SUL—0.06	1	−1.9	−2.3	**5.5**	**5.5**
		2	−2.1	−2.3	1.7	**5.4**
	ERT—0.25	1	−2.3	**5.0**	**5.0**	**5.0**
		2	−2.5	**5.0**	**5.0**	**5.0**
*E. coli* 937054	SUL—0.12	1	−2.2	−2.2	**5.1**	**5.1**
		2	−1.7	−1.8	**5.5**	**5.5**
	ERT—0.25	1	−1.7	2.0	**5.1**	**5.1**
		2	−0.3	**5.2**	**5.2**	**5.2**
*K. pneumoniae* ATCC 700603	SUL—0.25	1	−2.1	−2.2	−2.4	**5.3**
		2	−2.5	−2.6	−2.3	**5.1**
	ERT—0.12	1	−2.2	−2.2	−2.1	−2.1
		2	−2.2	−2.2	−2.1	−1.9
*K. pneumoniae* 396798	SUL—0.12	1	−2.0	**4.4**	**5.1**	**5.1**
		2	−1.9	**4.1**	**5.1**	**5.1**
	ERT—0.5	1	−2.0	**5.0**	**5.0**	**5.0**
		2	−1.7	**5.0**	**5.0**	**5.0**

^*a*^ The modal MIC value was determined from triplicate broth macrodilution testing.

^*b*^ Bold values represent a ≥3 log_10_ drop in CFUs compared to the starting inoculum. A negative number indicates higher CFUs compared to the starting inoculum. ERT, ertapenem; MIC, minimum inhibitory concentration; SUL, sulopenem.

**TABLE 6:** Summary of MIC and decrease in cell viability during the time-kill kinetics assay at the 8-hour time point for sulopenem and ertapenem against all isolates and strains^*b*^

Organism	Broth macrodilution MIC^*a*^ (mg/L)	Replication	Antimicrobial agent concentration (relative to MIC)			
			0.5×	2×	4×	8×
*E. coli* ATCC 25922	SUL—0.03	1	−2.2	1.5	2.2	**3.3**
		2	−2.3	1.8	2.1	**3.2**
	ERT— 0.015	1	**3.5**	**4.2**	**4.2**	**4.0**
		2	**3.1**	**3.9**	**3.9**	**4.6**
*E. coli* ATCC 35218	SUL—0.03	1	−1.6	2.2	2.3	2.5
		2	−1.6	2.2	2.1	2.5
	ERT—0.015	1	2.2	2.2	2.2	2.2
		2	2.3	2.5	2.4	2.4
*E. coli* NCTC 13353	SUL—.06	1	−1.5	1.3	**3.7**	**4.7**
		2	−1.5	1.0	**3.4**	**4.3**
	ERT—0.25	1	0.0	**5.0**	**5.0**	**5.0**
		2	0.8	**4.4**	**4.7**	**5.0**
*E. coli* 937054	SUL—0.12	1	−1.4	3.0	**5.1**	**5.1**
		2	−0.5	2.4	**4.4**	**5.2**
	ERT—0.25	1	−0.9	2.4	**4.6**	**5.1**
		2	−0.7	**4.6**	**5.2**	**5.2**
*K. pneumoniae* ATCC 700603	SUL—0.25	1	0.3	1.7	**3.3**	**5.3**
		2	0.5	0.9	2.6	**5.1**
	ERT—0.12	1	1.5	1.4	1.2	1.1
		2	1.3	1.3	1.5	0.5
*K. pneumoniae* 396798	SUL—0.12	1	2.0	**4.2**	**4.5**	**4.2**
		2	2.0	**3.9**	**4.6**	**4.5**
	ERT—0.5	1	−0.1	**4.6**	**4.3**	**5.0**
		2	0.2	**4.4**	**5.0**	**5.0**

^*a*^ The modal MIC value was determined from triplicate broth macrodilution testing.

^*b*^ Bold values represent a ≥3 log_10_ drop in CFUs compared to the starting inoculum. A negative number indicates higher CFUs compared to the starting inoculum. ERT, ertapenem; MIC, minimum inhibitory concentration; SUL, sulopenem.

**Fig 3:**
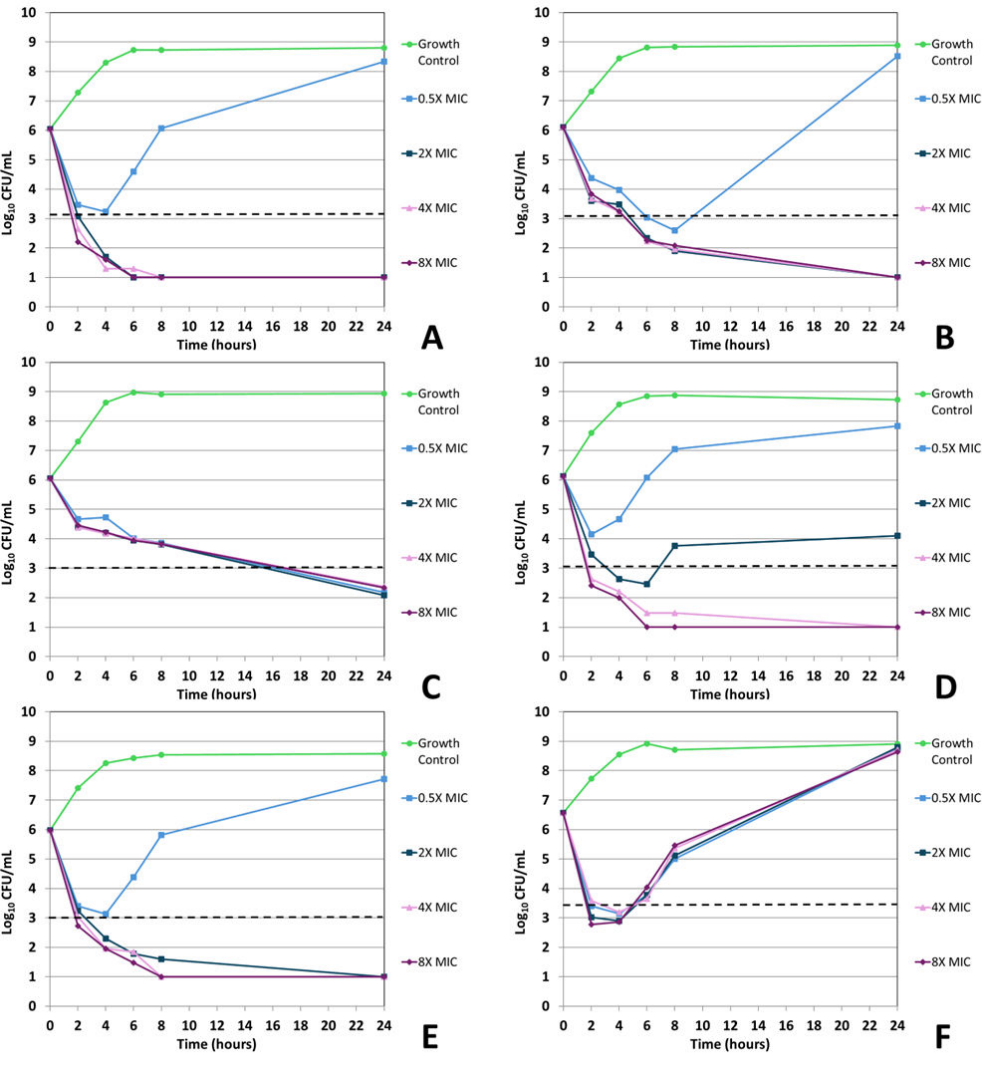
*In vitro* time-kill curve analysis of ertapenem against all isolates and strains. Time-kill curves for iteration 1 of ertapenem against *E. coli* NCTC 13353 (**A**), *E. coli* ATCC 25922 (**B**), *E. coli* ATCC 35218 (**C**), *E. coli* 937054 (**D**), *K. pneumoniae* 396798 (**E**), and *K. pneumoniae* ATCC 700603 (**F**). The dashed line indicates ≥3 log_10_ CFU/mL reduction in viable organism counts compared to the untreated growth control.

**Fig 4:**
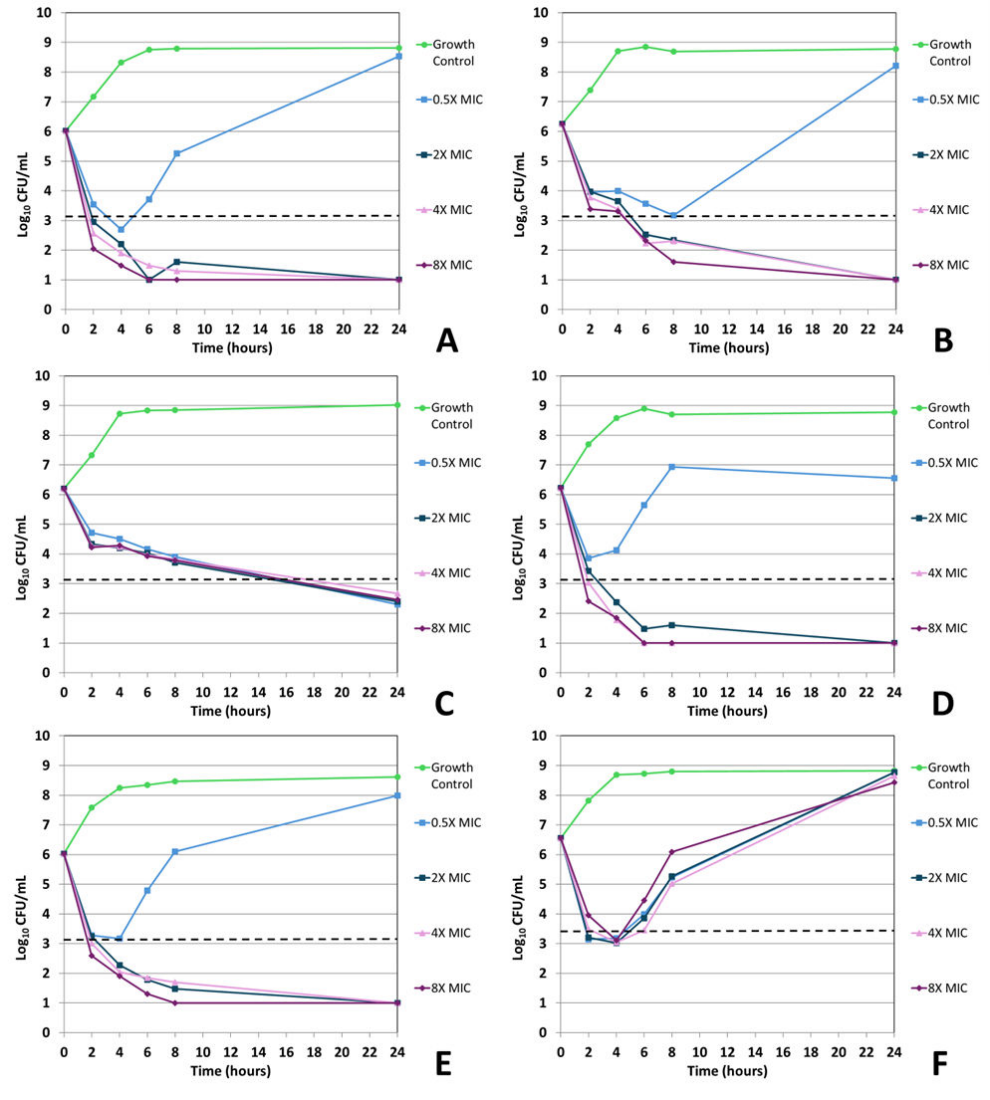
*In vitro* time-kill curve analysis of ertapenem against all isolates and strains. Time-kill curves for iteration 2 of ertapenem against *E. coli* NCTC 13353 (**A**), *E. coli* ATCC 25922 (**B**), *E. coli* ATCC 35218 (**C**), *E. coli* 937054 (**D**), *K. pneumoniae* 396798 (**E**), and *K. pneumoniae* ATCC 700603 (**F**). The dashed line indicates ≥3 log_10_ CFU/mL reduction in viable organism counts compared to the untreated growth control.

Ertapenem displayed bactericidal activity in most (5/6) ertapenem isolate time-kill assays when tested at 8× and 4× the baseline MIC concentration, achieving this activity (Fig. 3 and 4; Table 5) after 24 hours of incubation. At 0.5× the ertapenem MIC, bactericidal activity was seen in both time-kill replicates for the *E. coli* ATCC 35218 isolate; however, most time-kill studies using 0.5× ertapenem MIC showed an increase in CFUs following 24 hour incubation (0.3–2.5 log_10_ increase, Fig. 3 and 4). Of the 8× and 4× ertapenem time-kills displaying bactericidal activity (5/6), 4/6 were bactericidal within 8 hours (Tables 5 and 6).

For ertapenem and sulopenem, regrowth—defined as ≥1 log_10_ decrease in viable cells from the starting inoculum followed by a ≥2 log_10_ increase in viable cells above the starting inoculum—was recorded for all isolates/strains but *K. pneumoniae* 396798. When testing sulopenem, this phenomenon was seen in 937054—0.5× and 2.0× MIC; 25922—0.5×, 2.0×, and 4.0× MIC; 35218—0.5× MIC; 13353—2× MIC; and 700603—0.5×, 2.0×, and 4× MIC (Fig. 1 and 2). When testing ertapenem, this phenomenon was seen in 25922—0.5× MIC; 13353—0.5×; and 700603—0.5×, 2.0×, 4.0×, and 8.0× MIC (Fig. 3 and 4). *K. pneumoniae* ATCC 700603 was the test organism in the majority of time-kill assays displaying regrowth. Of the sulopenem and ertapenem time-kill assays exhibiting regrowth, 20 of the 26 replicates were at concentrations ± 1 log_2_ dilution of the broth macrodilution modal MIC (Fig. 1 to 4).

## DISCUSSION

No instances of antagonism between sulopenem and comparator agents (representing nine different antibiotic classes) were observed in checkerboard testing. Minimal synergy was observed when sulopenem was combined with trimethoprim-sulfamethoxazole against *E. coli* (ATCC 35218 and #937054) and when combined with gentamicin against *K. pneumoniae* (#396798). Indifference was the most common observation for the sulopenem checkerboard combinations, with 61.1% (33/54) having an FIC value of >0.5 to ≤4.0. Follow-up time-kill assays utilizing these agents alone and in combination were not completed; therefore, the potential clinical significance of the observed synergism remains unclear.

Against ATCC 25922, 700603, NCTC 13353, and the clinical isolate 937054, sulopenem displayed no PAE interval; against ATCC 35218 and the clinical isolate 396798, sulopenem displayed minimal PAE intervals (0.2–0.7 hours). Comparatively, ertapenem testing demonstrated longer PAE intervals against the two clinical isolates and ATCC 700603 (0.2–1.1 hours) and comparable PAEs for the remaining strains. These findings are consistent with PAE testing of other β-lactam agents against Gram-negative bacteria (14–16). The minimal PAE observed for sulopenem, in addition to an elimination half-life of 0.88–1.03 hours (1), suggests that sulopenem—similar to other carbapenem agents—requires repeated dosing to maintain drug concentrations above the MIC (17–19). Currently, sulopenem etzadroxil (the oral prodrug of sulopenem) is co-administered with probenecid, an orally available organic ion transport inhibitor that delays the excretion of sulopenem through the kidneys, twice daily as a fixed dose (500 mg/500 mg) combination bilayer tablet, while sulopenem is given intravenously as 1,000 mg once daily followed by oral step-down therapy (20). The pharmacodynamic attributes of this dosing regimen (sulopenem etzadroxil/probenecid 500 mg/500 mg [every 12 hours]) were recently evaluated under *in vitro* one-compartment and hollow-fiber infection models, with results demonstrating a reduction in bacterial density from 1.0 × 10^6^ CFU/mL to below 1 log_10_ CFU/mL over the study duration (21, 22). Following exposure to 5× the sulopenem MIC, on-scale PAE-SME intervals were recorded for most isolates when resuspended in 0.25× MIC concentrations, and off-scale intervals (>5 hours) were seen when challenged with 0.5× the MIC. While evaluating ertapenem, similar data were recorded with longer PAE-SME intervals when resuspended at 0.25× MIC (3.0–>4.8 hours) compared to 0.5× MIC (>4.8 hours). The observed PAE-SME intervals for both sulopenem and ertapenem were longer than PAE intervals, indicating that a longer PAE is achievable with sub-inhibitory sulopenem and ertapenem concentrations following exposure to a supra-inhibitory concentration. Additionally, PAE-SMEs could have been longer; however, measurements were only taken within an 8 hour sampling schedule. These PAE and PAE-SME results should help to inform further pharmacokinetic study on the absorption, distribution, and elimination of this agent *in vivo*.

Ertapenem time-kill data corroborate previous reports (23–25) with regrowth observed at test concentrations ± 1 dilution of the MIC (26). Several sulopenem and ertapenem time-kill conditions resulted in regrowth, a phenomenon that agrees with published reports (26–28). Most conditions yielding regrowth were ±1 dilution of the baseline MIC and all contained selection concentrations of 1–0.008 mg/L. The implication of these findings regarding clinical efficacy is not well understood, as *in vitro* time-kill methodology contains a single input (or “dose”) of antibiotic with cell viability measured over a 24 hour time course. Previous reports have shown bactericidal activity of sulopenem when tested against Gram-positive and Gram-negative bacterial isolates (29, 30). Of note, sulopenem sustained bactericidal activity throughout time-kill kinetic assays against a selection of β-lactamase producing Gram-negative bacteria, including those producing an ESBL (Table 2). Bactericidal activity of sulopenem was observed against all six isolates under at least 1× MIC test condition. This activity was seen at 8×, 4×, and 2× baseline MICs, in many cases occurring earlier than the terminal 24 hour CFU sampling point. Additional time-kill studies evaluating sulopenem *in vitro* activity with trimethoprim-sulfamethoxazole and gentamicin individually and in combination would provide confirmation of the observed synergy results and illustrate additional applications of this compound.

In summary, sulopenem activity was characterized using *in vitro* time-kill, PAE, and PAE-SME methods against a collection of β-lactamase-producing Gram-negative clinical isolates and quality control strain species commonly found in patients with urinary tract infection. Minimal PAE intervals were observed for sulopenem, and bactericidal activity was seen in at least one test condition against all six isolates/strains studied. No instances of antagonism were observed in checkerboard testing, and minimal synergistic interactions were observed when sulopenem was tested in combination with comparator agents. The clinical implication of these findings is not well understood. However, the oral dosing regimen of sulopenem etzadroxil/probenecid 500 mg/500 mg administered every 12 hours was recently evaluated in two phase 3 clinical trials where sulopenem demonstrated efficacy in comparison to amoxicillin-clavulanate in uUTI and against ciprofloxacin in fluoroquinolone-resistant uUTI. Additionally, more work should be completed to further characterize the *in vitro* activity of this agent. Several reports detailing sulopenem activity against *Mycobacterium* spp. and anaerobes (3, 8, 31) suggest the need for future investigation into the potential utility of this agent against non-target species or indications ancillary to the treatment of complicated and uncomplicated urinary tract infections.

## MATERIALS AND METHODS

### Isolates and strains

Synergy, PAE, and time-kill testing included the following quality control strains: *Escherichia coli* ATCC 25922, *E. coli* ATCC 35218, *E. coli* NCTC 13353, and *Klebsiella pneumoniae* ATCC 700603. In addition, molecularly characterized clinical isolates from the SENTRY Antimicrobial Surveillance Program (*E. coli* #937054 [CTX-M-15] and *K. pneumoniae* #396798 [CTX-M-15, SHV-1, and OXA-1/30]) were included based on their ESBL genotype (Table 4) (32). Isolates were PCR screened using primers targeting ESBL genes. Amplicons were sequenced on both strands and the nucleotide sequences, and deduced amino acid sequences were analyzed using the Lasergene software package (DNASTAR, Madison, WI, USA). Sequences were compared to those available via internet sources (33). Table 3 displays an isolate antibiogram.

### Checkerboard testing

To evaluate for synergy, indifference, or antagonistic interactions, sulopenem and nine antimicrobial agents representing separate and distinct drug classes were tested against all bacterial isolates and strains via checkerboard testing methodology. The agents tested include amoxicillin, aztreonam, ceftriaxone, doxycycline, gentamicin, levofloxacin, nitrofurantoin, trimethoprim-sulfamethoxazole, and vancomycin. Checkerboard panel preparation included the use of cation-adjusted Mueller-Hinton broth (CAMHB) media and followed previously described methods (13). The fractional inhibitory concentration (ΣFIC) was calculated for each antibiotic combination using the following formula: ΣFIC = FICA + FICB (34). In this formula, FICA equals the MIC of agent A in combination divided by the MIC of agent A alone, while FICB refers to the MIC of agent B in combination divided by the MIC of agent B alone. The ΣFIC_min_ and ΣFIC_max_ denote the minimum and maximum ΣFIC calculated from the checkerboard synergy panel. Categorical interpretation of sulopenem checkerboard combinations were defined as synergy when the ΣFIC was ≤0.5, indifferent when the ΣFIC was >0.5 to ≤4.0, and antagonistic when the ΣFIC was >4.0. In checkerboard combinations where one of the comparator agent MIC values was greater than the highest concentration evaluated, no ΣFIC was calculated.

### Post-antibiotic and sub-MIC effect assay

Sulopenem and ertapenem modal baseline MIC values were determined from triplicate broth microdilution susceptibility testing for all six bacterial isolates or strains. Testing utilized CAMHB media, frozen-form 96-well panels, and Clinical and Laboratory Standard Institute reference methodology (35, 36). The modal MIC value selected during broth microdilution testing determined the MIC multiples used in PAE and PAE-SME testing.

PAE and PAE-SME testing were conducted following established methodology (12, 37). The PAE is defined as the time interval of bacterial growth suppression that occurs following a short exposure to an antimicrobial agent, while PAE-SME is the time interval of bacterial growth suppression that occurs following removal of the initial antimicrobial agent and reintroducing a sub-inhibitory concentration of the same agent. For PAE testing, the *E. coli* and *K. pneumoniae* isolates were exposed to sulopenem or ertapenem at 1×, 5×, and 10× the modal broth microdilution MIC value for a period of 1 hour. For PAE-SME testing, only the initial 5× antimicrobial agent concentration was used, followed by removal of the antibiotic via dilution (1:1,000) and reintroducing sub-inhibitory (0.25× MIC and 0.5× MIC) concentrations. The 1× and 10× MIC concentrations were only used for PAE testing. PAE and PAE-SME testing was conducted using CAMHB. Bacterial colony counts were taken at *T*_0_ (pre-exposure), before and after the dilution step (*T*_1A_ and *T*_1B_), and at every subsequent timepoint (*T*_2_ – *T*_8_). Counts were graphed, and the PAE or PAE-SME durations for sulopenem and ertapenem were calculated. PAE was calculated using the formula outlined in Craig and Gudmundsson: PAE = *T* – *C*, where *T* is the time required for the CFU per milliliter of the test culture to increase 1 log_10_ above the count observed immediately following drug removal, and *C* is the corresponding time for the untreated control culture (19). PAE-SME was calculated using the formula described in Cars and Odenholt-Tornqvist: PAE-SME = *M* – *C*, where *M* is the time required for the CFU per milliliter in the sub-MIC test culture to increase 1 log_10_ above the count observed following drug removal/addition of the sub-MIC concentration, and *C* is the corresponding time for the untreated control culture (12).

### *In vitro* time-kill assay

The four *E. coli* and two *K. pneumoniae* isolates and quality control strains were tested via *in vitro* time-kill methodology (13) in duplicate. Time-kill testing utilized CAMHB test media with a starting inoculum of approximately 5 × 10^5^ CFU/mL and sulopenem and ertapenem antimicrobial concentrations of 0.5×, 2.0×, 4.0×, and 8.0× the broth macrodilution MIC value. Time-kill tubes were sampled in CFU per milliter at *T*_0_ (0 hour), *T*_2_, *T*_4_, *T*_6_, *T*_8_, and *T*_24_, and the bactericidal activity was defined as ≥3 log_10_ CFU/mL reduction in viable organism counts compared with the untreated growth control (13).

## Data Availability

Data generated for this study will be made available upon request.
